# Association of preoperative anxiety and depression with quality of recovery after caesarean delivery: a prospective observational study

**DOI:** 10.1186/s40981-025-00782-z

**Published:** 2025-03-29

**Authors:** Ayu Ishida, Mitsuru Ida, Akane Kinomoto, Yusuke Naito, Masahiko Kawaguchi

**Affiliations:** 1 Department of Perioperative Management Center, Nara Medical University Hospital, 840 Shijocho, Kashihara, Nara 634-8522 Japan; 2 Department of Anesthesiology, Nara Medical University, 840 Shijocho, Kashihara, Nara 634-8521 Japan

**Keywords:** Anxiety, Caesarean delivery, Depression, Patient-reported outcome measures

## Abstract

**Introduction:**

To investigate the association between the presence of both anxiety and depression and the quality of recovery after caesarean delivery.

**Methods:**

This secondary analysis of a prospective observational study included 137 patients aged ≥ 18 years who underwent elective and urgent caesarean delivery under spinal anesthesia and completed the Obstetric Quality of Recovery-11 scale at least once postoperatively. Before caesarean delivery, patients were screened for anxiety and depression using the Hospital Anxiety and Depression Scale. A total score of ≥ 8 in each subscale was considered positive screening. Postpartum quality of recovery was assessed using the Obstetric Quality of Recovery-11 at three time points, with a higher score indicating better recovery.

**Results:**

Among the eligible patients, 17.5% (24/137) screened positive for both anxiety and depression. No significant difference was found in the Obstetric Quality of Recovery-11 score 24 h after caesarean delivery (*p* = 0.13, Cohen’s d = 0.33), but differences were observed on postoperative day 3 (*p* = 0.004, Cohen’s d = 0.67) and postoperative day 5 (*p* = 0.01, Cohen’s d = 0.58). In the multiple regression analysis, after adjusting for prominent clinical factors, the presence of both anxiety and depression was associated with the Obstetric Quality of Recovery-11 score on postoperative day 3 (*p* = 0.01) and postoperative day 5 (*p* = 0.01), but not 24 h after delivery (*p* = 0.19).

**Conclusions:**

Positive Hospital Anxiety and Depression Scale screening for both anxiety and depression was associated with a poor quality of recovery, measured using the Obstetric Quality of Recovery-11 scores on PODs 3 and 5.

**Supplementary Information:**

The online version contains supplementary material available at 10.1186/s40981-025-00782-z.

## Introduction

The number of patients undergoing caesarean delivery is increasing every year, and one in three to five pregnant women in Japan and the United States gives birth via caesarean section [1, 2]. Although pain intensity and opioid consumption are the most studied short-term outcomes after caesarean delivery [3], opioid use to mitigate pain can cause nausea, vomiting, and dizziness. Additionally, the experience of caring for a newborn should be focused on after caesarean delivery. To address these issues, the Obstetric Quality of Recovery-11 (ObsQoR-11), which includes maternal recovery and the ability to care for newborns, was developed and evaluated for patients who had undergone caesarean delivery in 2019 [4]; however, no factors associated with postpartum quality of recovery have been studied, except for delivery modes [5–8].

Anxiety and depression are the most common mental disorders during pregnancy, with prevalence rates of approximately 20% each [9–11]. Pre-existing mental health disorders and acute pain after caesarean delivery are associated with postpartum depression [12–15]; however, no evidence suggests that anxiety and depression during pregnancy affect the immediate ObsQoR after caesarean delivery.

We hypothesized that patients with both anxiety and depression before caesarean delivery would be more likely to have a lower ObsQoR-11 score and aimed to investigate the association of anxiety and depression with the quality of recovery after caesarean delivery.

## Methods

This planned secondary analysis of an prospective observational study was approved by the Nara Medical University Institutional Review Board, Kashihara, Nara, Japan (Chairperson Prof. M. Yoshizumi; Approval No. 3051 on March 9, 2023). Our initial study aimed to assess the validity of the ObsQoR-11 and evaluate its association with postoperative depression and functional disability [16]. Written informed consent was obtained from all the patients. This study was conducted according to the principles of the Declaration of Helsinki.

### Study population

Our initial study included patients aged 18 years or older who underwent elective and urgent caesarean delivery under spinal anesthesia at Nara Medical University between April 2023 and January 2024, which was predicted to be completed from 7:00 a.m. to 7:00 p.m. We excluded patients who underwent emergency caesarean delivery such as (categories 1 and 2 shown in the National Institute for Health and Care Excellence guideline) and those who had no sufficient time for the explanation of this study.^24^ Other exclusion criteria were as follows: patients who were not fluent in Japanese, patients with psychiatric illnesses, patients who had epidural labor before caesarean delivery, and patients who converted from spinal anesthesia to general anesthesia. Patients who answered the ObsQoR-11 at least once postoperatively were included in the analysis.

### Data collection

We evaluated patient characteristics, including age, height, weight, drinking, smoking status, gestational age, presence of a partner, previous caesarean delivery, treatment for infertility, gravidity, anxiety and depression, number of fetuses, hypertensive disorders of pregnancy, gestational diabetes, thyroid disease, ritodrine administration, magnesium infusion, and urgent delivery. Intraoperative data, including surgical duration and intraoperative blood loss, were also collected. Anxiety and depression were assessed using the Hospital Anxiety and Depression Scale (HADS), originally developed by Zigmond and Snaith in 1983 [17, 18]. The HADS was designed to detect a possibility of anxiety and depression in hospital settings and consists of 14 items, with seven items assessing anxiety (HADS-A) and seven items assessing depression (HADS-D). Each item is scored on a 4-point Likert scale (0 to 3), resulting in a total score ranging from 0 to 21 for each subscale. A score of ≥ 8 in either subscale is commonly used to indicate clinically relevant symptoms of anxiety or depression. HADS has been validated in various patient populations and is widely used in clinical and research settings. Although it was not specifically developed for pregnant women, numerous studies have employed HADS as a screening tool in this population [19–21].

Patients undergoing elective caesarean delivery were informed of the study the day before, and patients undergoing urgent caesarean delivery were informed of the study and consent was obtained at the time of the decision to have a caesarean delivery, and the patient’s characteristics were assessed.

### Perioperative management

Our institution performed perioperative management in accordance with our local protocol. Pregnant women undergoing scheduled caesarean deliveries were allowed to drink water for up to 2 h before entering the operating room. In the case of urgent surgery, patients were unable to eat or drink anything once the surgery was scheduled. In the operating room, spinal anesthesia is administered with 2–2.5 mL hyperbaric bupivacaine (0.5%), 10 μg fentanyl, and 100 μg morphine with the patient in the lateral position after attaching the standard anesthesia monitors. Intraoperative patient management, including blood pressure management, additional sedation, and analgesia, was performed at the discretion of the anesthesiologist in charge of the patient. After returning to the general maternity ward, acetaminophen was administered every 6 h for up to 24 h after delivery, followed by oral analgesics as needed. Obstetricians and midwives provided the remaining management in the obstetric wards according to their protocols.

### Outcomes

The primary outcome in this study was the ObsQoR-11 score, which was assessed at 24 h, 3 days, and 5 days after caesarean delivery. The ObsQoR-11 was developed in 2019 and consists of 11 items: pain (moderate and severe), vomiting, dizziness, shivering, comfort, morbidity, taking care of the newborn (holding and feeding), hygiene, and feeling in control [4]. The total score ranged from 0 to 110, with higher scores indicating better recovery [4]. The validity of Japanese version has been confirmed in our initial study [16].

### Statistical analysis

Continuous data are presented as the mean (standard deviation) and categorical variables as numbers (percentage) [22]. Data from the two groups were compared using the chi-squared test or Fisher’s exact test for dichotomous variables and an unpaired t-test for continuous variables. The unadjusted and adjusted partial regression coefficient for the presence of both anxiety and depression for the ObsQoR-11 was calculated using multiple regression analysis, adjusting for maternal age, smoking, gestational age, presence of a partner, previous caesarean delivery, treatment for infertility, number of fetuses, hypertensive disorders of pregnancy, gestational diabetes, thyroid disease, urgent caesarean delivery, surgical duration, and intraoperative blood loss. As a sensitivity analysis, outcomes were compared in a similar fashion for patients with and without anxiety and for patients with and without depression. SPSS version 25.0 (IBM Inc., Armonk, NY, USA) was used for all analyses, and statistical significance was set at *p* < 0.05. The sample size was not calculated and was dependent on our initial study. Thus, Cohen’s d effect size was calculated (0.1, small effect size; 0.3, moderate effect size; and 0.5, large effect size) [23].

## Results

Of the 150 eligible patients in our original study, 137 answered the ObsQoR-11 at least once postoperatively, and 24 (17.5%) were likely to have both anxiety and depression. Regarding patient demographics and intraoperative data, as shown in Table 1, gestational age (37.5 weeks vs. 36.0 weeks, *p* = 0.03) was significantly different between those with negative and positive screening (respectively) for anxiety and depression. Table 2 shows the ObsQoR-11 scores at three points after delivery. There was no significant difference in the ObsQoR-11 score 24 h after delivery (*p* = 0.13, Cohen’s d = 0.33); however, differences were observed on postoperative day (POD) 3 (*p* = 0.004, Cohen’s d = 0.67) and POD 5 (*p* = 0.01, Cohen’s d = 0.58). In the multiple regression analysis, even after adjusting for clinically prominent factors, the likelihood of the presence of both anxiety and depression before delivery was associated with the ObsQoR-11 score on POD 3 (*p* = 0.01) and POD 5 (*p* = 0.01), but not at 24 h after delivery (*p* = 0.19) (Table 3). One patient who drank during pregnancy also smoked; thus, only drinking was adjusted in the multiple regression analysis. No significant differences were between patients with and without anxiety and between patients with and without depression (Supplementary Tables 1–3).

**Table 1:** Patient demographics and intraoperative data

	Patients with a negative screening for anxiety and/or depression * (n = 113)	Patients with a positive screening for both anxiety and depression (n = 24)	P-value
Age (year), mean (SD)	34.1 (5.5)	33.5 (5.4)	0.58
Height (cm), mean (SD)	157.5 (4.9)	157.9 (5.1)	0.72
Weight (kg), mean (SD)	65.4 (11.4)	61.3 (8.2)	0.10
Drinking during pregnancy, number (%)	6 (5.3)	2 (8.3)	0.62
Smoking during pregnancy, number (%)	1 (0.08)	0 (0.0)	> 0.99
Gestational age (week), mean (SD)	37.5 (2.9)	36.0 (3.6)	0.03
Presence of partner, number (%)	108 (95.6)	21 (87.5)	0.14
Previous caesarean section, number (%)			0.38
0	58 (51.3)	16 (66.7)	
1	43 (38.1)	6 (25.0)	
2	12 (10.6)	2 (8.3)	
Treatment for infertility, number (%)	32 (28.0)	8 (33.3)	0.60
Gravid (n), mean (SD)	2.2 (1.3)	1.7 (1.1)	0.10
HADS score			
Anxiety, mean (SD)	4.7 (2.6)	10.7 (2.8)	< 0.001
Depression, mean (SD)	4.4 (2.5)	9.9 (1.9)	< 0.001
Number of fetuses, number (%)			0.06
Single	98 (86.7)	17 (70.8)	
Twin	15 (13.3)	7 (29.2)	
Hypertensive disorders of pregnancy, number (%)	10 (8.8)	3 (12.5)	0.70
Gestational diabetes, number (%)	7 (6.2)	1 (4.2)	> 0.99
Thyroid disease, number (%)	8 (7.1)	2 (8.3)	0.69
Ritodrine administration, number (%)	17 (15.0)	7 (29.2)	0.13
Magnesium infusion, number (%)	21 (18.6)	7 (29.2)	0.26
Urgent surgery, number (%)	45 (39.8)	12 (50.0)	0.49
Surgical duration (min), mean (SD)	62.3 (18.5)	54.3 (19.4)	0.05
Intraoperative blood loss (mL), mean (SD)	599 (357)	571 (265)	0.72

*SD* standard deviation, *HADS* Hospital Anxiety and Depression Scale

^*^This group included the following patients: i) anxiety score < 8 and depression score < 8 (no anxiety or depression), ii) anxiety score ≥ 8 and depression score < 8 (anxiety but no depression), iii) anxiety score < 8 and depression score ≥ 8 (depression but no anxiety)

**Table 2:** Obstetric Quality of Recovery-11 score

	Patients with a negative screening for anxiety and/or depression (*n* = 113)	Patients with a positive screening for both anxiety and depression (*n* = 24)	*P* value	Cohen's d
24 h, mean (SD)	68.0 (17.3) (*n* = 110)	61.8 (19.5) (*n* = 24)	0.13	0.33
POD 3, mean (SD)	90.1 (10.2) (*n* = 111)	83.3 (15.0) (*n* = 22)	0.004	0.67
POD 5, mean (SD)	97.2 (9.3) (*n* = 104)	91.0 (12.3) (*n* = 22)	0.01	0.58

*SD* standard deviation, *POD* postoperative day

**Table 3:** Results of multiple regression analysis

	Partial regression coefficient (95% confidence interval)	*P*-value	Adjusted partial regression coefficient (95% confidence interval)	*P*-value
24 h (n = 134)	−6.1 (−14.2, 1.94)	0.13	−5.7 (−14.2, 2.8)	0.19
POD 3 (n = 133)	−7.7 (−12.9, −2.5)	0.004	−6.1 (−11.3, −1.06)	0.01
POD 5 (n = 126)	−6.0 (−10.6, −1.3)	0.01	−6.1 (−11.3, −0.8)	0.01

*POD* postoperative day. In the adjusted model, age, smoking, gestational age, presence of a partner, previous caesarean section, infertility treatment, number of fetuses, hypertensive disorders of pregnancy, gestational diabetes, thyroid disease, urgent caesarean section, surgical duration, and intraoperative blood loss were adjusted

## Discussion

This planned secondary analysis revealed that 17.5% of patients had a likelihood of having both anxiety and depression screened using the HADS before caesarean delivery, and they had lower ObsQor-11 scores on PODs 3 and 5.

In one nationwide study conducted in Italy and one meta-analysis, prenatal anxiety and depression were assessed using independent screening tools: the Edinburgh Postnatal Depression Scale and the General Anxiety Disorder scale [9, 10]. In contrast, this study adopted the HADS, which is a validated tool that can evaluate both anxiety and depression using a single tool to reduce the burden and time on patients. It is well-known that prepartum depression is a strong predictor for postpartum depression [12–14]; however, the association between prepartum anxiety and depression and short-term outcomes has been poorly documented, and this study revealed these associations. The *p-*value for the presence of both anxiety and depression for the ObsQoR-11 score at 24 h after caesarean delivery exceeded 0.05, but the effect size was moderate to large, suggesting a small sample size. Conversely, a large effect size on PODs 3 and 5 was observed. The results of the multiple regression analyses showed similar trends. The ObsQoR-11 score at 24 h after caesarean delivery might be more affected by delivery and anesthesia than at PODs 3 and 5, and may be less reflective of individual mental health. Sensitivity analyses assessing the impact of anxiety or depression on the ObsQoR did not yield statistically significant differences. Although both are often lumped together as mood factors that affect postoperative outcomes, anxiety and depression have different effects on postoperative outcomes [24]. The finding from this study that the presence of both, rather than individually, affects the ObsQoR is an important finding and leads to the merit of using a measure that can assess both anxiety and depression, such as the HADS.

Some previous studies have shown the reliability of the ObsQoR-10 and ObsQoR-11 [7, 8, 25–28], but no studies have investigated the factors associated with the ObsQoR-11, except for modes of delivery that are difficult to modify [5–8]. Our results highlight the importance of assessing and managing prenatal mental health status. Furthermore, preoperative anxiety can be potentially modified using mild exercises such as yoga and personalized techniques for reducing anxiety [29, 30]. In this study, mental status was assessed at one day (elective caesarean) or several hours (urgent caesarean) before delivery; thus, the duration of the intervention was limited. Further studies are needed to examine the duration of intervention required to increase the ObsQoR-11 score. In this cohort, pregnant women with a positive screening for both anxiety and depression had shorter gestational age. This can be explained by the fact that poor mental health lead to higher corticotropin-releasing hormone and cortisol levels, which are likely to lead to preterm delivery [31]. Then, these higher stress hormone levels may have reduced the quality of recovery and should be evaluated in future studies.

This study has several limitations. First, the generalizability of our findings may be limited because this was a single-center study conducted in Japan. Second, this study cannot present a causal relationship between preoperative anxiety and depression and poor postoperative recovery. However, our findings further emphasize the importance of a preoperative mental health assessment. Third, in this study, the HADS was selected to assess maternal mental status; however, the use of different assessment tools might lead to different results. Fourth, because maternal mental health was screened in the hospital prior to caesarean delivery, it may not be possible to distinguish between symptoms in the third trimester of pregnancy or prior to caesarean delivery.

## Conclusion

The presence of both anxiety and depression, screened using the HADS, was associated with a poor quality of recovery, as measured using the ObsQoR-11 on PODs 3 and 5. This study highlights the potential value of assessing maternal mental health before caesarean delivery to identify women at risk for poor recovery. Future studies including a larger sample size are required to investigate effects of anxiety and depression on postpartum recovery.

## Supplementary Information

Supplementary Material 1.

## Data Availability

Data supporting the findings of this study are available from the corresponding author upon request.

## Abbreviations

ObsQoR-11: Obstetric Quality of Recovery-11
HADS: Hospital Anxiety and Depression Scale
POD: Postoperative day
