# Supplementary material for: Association of preoperative anxiety and depression with quality of recovery after caesarean delivery: a prospective observational study
Source: JA Clin Rep. 2025 Mar 29;11:19. doi: 10.1186/s40981-025-00782-z (PMC11954767; doi:10.1186/s40981-025-00782-z)
Supplement: Supplementary file 1 — Supplementary Material 1. [file 40981_2025_782_MOESM1_ESM.docx]

Supplementary Table 1a. Patient demographics and intraoperative data between patients with or without a positive screening for anxiety

|  | Patients without anxiety positive (n=96) | Patients with anxiety (n=41) | P value |
| --- | --- | --- | --- |
| Age (year), mean (SD) | 34.0 (5.3) | 34.1 (6.1) | 0.93 |
| Height (cm), mean (SD) | 157.3 (4.8) | 158.0 (5.2) | 0.45 |
| Weight (kg), mean (SD) | 64.9 (11.3) | 64.0 (10.3) | 0.65 |
| Drinking during pregnancy, number (%) | 6 (6.2) | 2 (4.9) | > 0.99 |
| Smoking during pregnancy, number (%) | 1 (1.0) | 0 (0.0) | > 0.99 |
| Gestational age (week), mean (SD) | 37.3 (3.1) | 37.3 (3.1) | 0.48 |
| Presence of partner, number (%) | 92 (95.8) | 37 (90.2) | 0.24 |
| Previous caesarean section, number (%) | |  | 0.38 |
| 0 | 48 (50.0) | 26 (63.4) |  |
| 1 | 37 (38.5) | 12 (29.3) |  |
| 2 | 11 (11.5) | 3 (7.3) |  |
| Treatment for infertility, number (%) | 26 (27.1) | 14 (34.1) | 0.41 |
| Gravid (n), mean (SD) | 2.3 (1.3) | 1.8 (1.1) | 0.05 |
| HADS score | 3.8 (1.7) | 10.2 (2.4) | < 0.001 |
| Anxiety, mean (SD) | 4.3 (2.6) | 7.7 (3.1) | < 0.001 |
| Depression, mean (SD) | |  | 0.45 |
| Number of fetuses, number (%) | 92 (85.4) | 33 (80.4) |  |
| Single | 14 (14.6) | 8 (19.5) |  |
| Twin | 10 (10.4) | 3 (7.3) | 0.75 |
| Hypertensive disorders of pregnancy, number (%) | 6 (6.2) | 2 (4.9) | > 0.99 |
| Gestational diabetes, number (%) | 5 (5.3) | 5 (12.2) | 0.16 |
| Thyroid disease, number (%) | 12 (12.5) | 12 (29.3) | 0.02 |
| Ritodrine administration, number (%) | 19 (19.8) | 9 (22.0) | 0.81 |
| Magnesium infusion, number (%) | 41 (42.7) | 16 (39.0) | 0.71 |
| Urgent surgery, number (%) | 63.2 (18.6) | 55.6 (18.4) | 0.03 |
| Surgical duration (min), mean (SD) | 607 (376) | 564 (246) | 0.50 |

SD: standard deviation, HADS: Hospital Anxiety and Depression Scale.

Supplementary Table 1b. Patient demographics and intraoperative data between patients with or without a positive screening for depression

|  | Patients without depression positive (n=101) | Patients with depression positive(n=36) | P value |
| --- | --- | --- | --- |
| Age (year), mean (SD) | 34.3 (5.5) | 33.4 (5.6) | 0.41 |
| Height (cm), mean (SD) | 157.5 (4.8) | 157.8 (5.3) | 0.76 |
| Weight (kg), mean (SD) | 65.5 (11.6) | 62.3 (9.1) | 0.14 |
| Drinking during pregnancy, number (%) | 6 (5.9) | 2 (5.6) | 1 |
| Smoking during pregnancy, number (%) | 1 (1.0) | 0 (0.0) | 1 |
| Gestational age (week), mean (SD) | 37.5 (3.0) | 36.4 (3.3) | 0.06 |
| Presence of partner, number (%) | 96 (95.0) | 33 ( 91.7) | 0.43 |
| Previous caesarean section, number (%) | |  | 1 |
| 0 | 54 (53.5) | 20 (55.6) |  |
| 1 | 36 (35.6) | 13 (36.1) |  |
| 2 | 11 (10.9) | 3 (8.3) |  |
| Treatment for infertility, number (%) | 29 (28.7) | 11 (30.6) | 0.83 |
| Gravid (n), mean (SD) | 2.2 (1.3) | 1.9 (1.2) | 0.25 |
| HADS score | 4.6 (2.7) | 8.8 (3.7) | < 0.001 |
| Anxiety, mean (SD) | 3.8 (1.9) | 9.7 (1.8) | < 0.001 |
| Depression, mean (SD) | |  | 0.l0 |
| Number of fetuses, number (%) | 89 (88.1) | 26 (72.2) |  |
| Single | 12 (11.9) | 10 (27.8) |  |
| Twin | 9 (8.9) | 4 (11.1) | 0.74 |
| Hypertensive disorders of pregnancy, number (%) | 6 (5.9) | 2 (5.6) | 1 |
| Gestational diabetes, number (%) | 6 (5.9) | 4 (11.1) | 0.45 |
| Thyroid disease, number (%) | 16 (15.8) | 8 (22.2) | 0.44 |
| Ritodrine administration, number (%) | 19 (18.8) | 9 (25.0) | 0.47 |
| Magnesium infusion, number (%) | 40 (39.6) | 17 (47.2) | 0.43 |
| Urgent surgery, number (%) | 62.5 (18.9) | 56.3 (18.1) | 0.09 |
| Surgical duration (min), mean (SD) | 608 (369) | 554 (251) | 0.41 |

SD: standard deviation, HADS: Hospital Anxiety and Depression Scale.

Supplementary Table 2a. Obstetric Quality of Recovery-11 score between patients with or without a positive screening for anxiety

|  | Patients without a positive screening for anxiety | Patients with a positive screening for anxiety | P value | Cohen's d |
| --- | --- | --- | --- | --- |
| 24 h, mean (SD) | 67.6 (17.8) (n=93) | 65.2 (19.2) (n=41) | 0.48 | 0.12 |
| POD 3, mean (SD) | 90.0 (10.7) (n=94) | 85.8 (12.9) (n=39) | 0.05 | 0.36 |
| POD 5, mean (SD) | 96.9 (9.5) (n=88) | 93.5 (11.4) (n=38) | 0.08 | 0.32 |

SD: standard deviation, POD: postoperative day.

Supplementary Table 2b. Obstetric Quality of Recovery-11 score between patients with or without a positive screening for depression

|  | Patients without a positive screening for depression | Patients with a positive screening for depression | P value | Cohen's d |
| --- | --- | --- | --- | --- |
| 24 h, mean (SD) | 68.0 (18.2) (n=98) | 63.8 (18.0) (n=36) | 0.23 | 0.25 |
| POD 3, mean (SD) | 89.4 (9.9) (n=99) | 87.0 (15.1) (n=34) | 0.30 | 0.18 |
| POD 5, mean (SD) | 97.0 (9.5) (n=93) | 93.0 (11.7) (n=33) | 0.05 | 0.37 |

SD: standard deviation, POD: postoperative day.

Supplementary Table 3a. Results of multiple regression analysis between patients with or without a positive screening for anxiety

|  | Partial regression coefficient (95% confidence interval) | P-value | Adjusted partial regression coefficient (95% confidence interval) | P-value |
| --- | --- | --- | --- | --- |
| 24 h (n=134) | -2.3 (-9.1, 4.4) | 0.48 | -2.6 (-9.7, 4.5) | 0.47 |
| POD 3 (n=133) | -4.2 (-8.5, 0.03) | 0.05 | -3.2 (-7.4, 0.9) | 0.12 |
| POD 5 (n=126) | -3.4 (-7.3, 0.4) | 0.08 | -3.0 (-7.0, 0.8) | 0.12 |

POD: postoperative day.

Supplementary Table 3a. Results of multiple regression analysis between patients with or without a positive screening for depression

|  | Partial regression coefficient (95% confidence interval) | P-value | Adjusted partial regression coefficient (95% confidence interval) | P-value |
| --- | --- | --- | --- | --- |
| 24 h (n=134) | -4.2 (-11.2, 2.8) | 0.23 | -3.3 (-10.8, 4.0) | 0.37 |
| POD 3 (n=133) | -2.3 (-6.9, 2.1) | 0.3 | -1.6 (-6.0, -2.8) | 0.47 |
| POD 5 (n=126) | -4.6 (-8.0, 0.06) | 0.05 | -3.5 (-7.6, 0.5) | 0.09 |

POD: postoperative day. In both adjusted models, age, smoking, gestational age, presence of a partner, previous cesarean section, infertility treatment, number of fetuses, hypertensive disorders of pregnancy, gestational diabetes, thyroid disease, urgent cesarean section, surgical duration, and intraoperative blood loss were adjusted.
